# Supplementary figures and images for: Potential of eye-tracking simulation software for analyzing landscape preferences
Source: PLoS One. 2022 Oct 27;17(10):e0273519. doi: 10.1371/journal.pone.0273519 (PMC9612490; doi:10.1371/journal.pone.0273519)

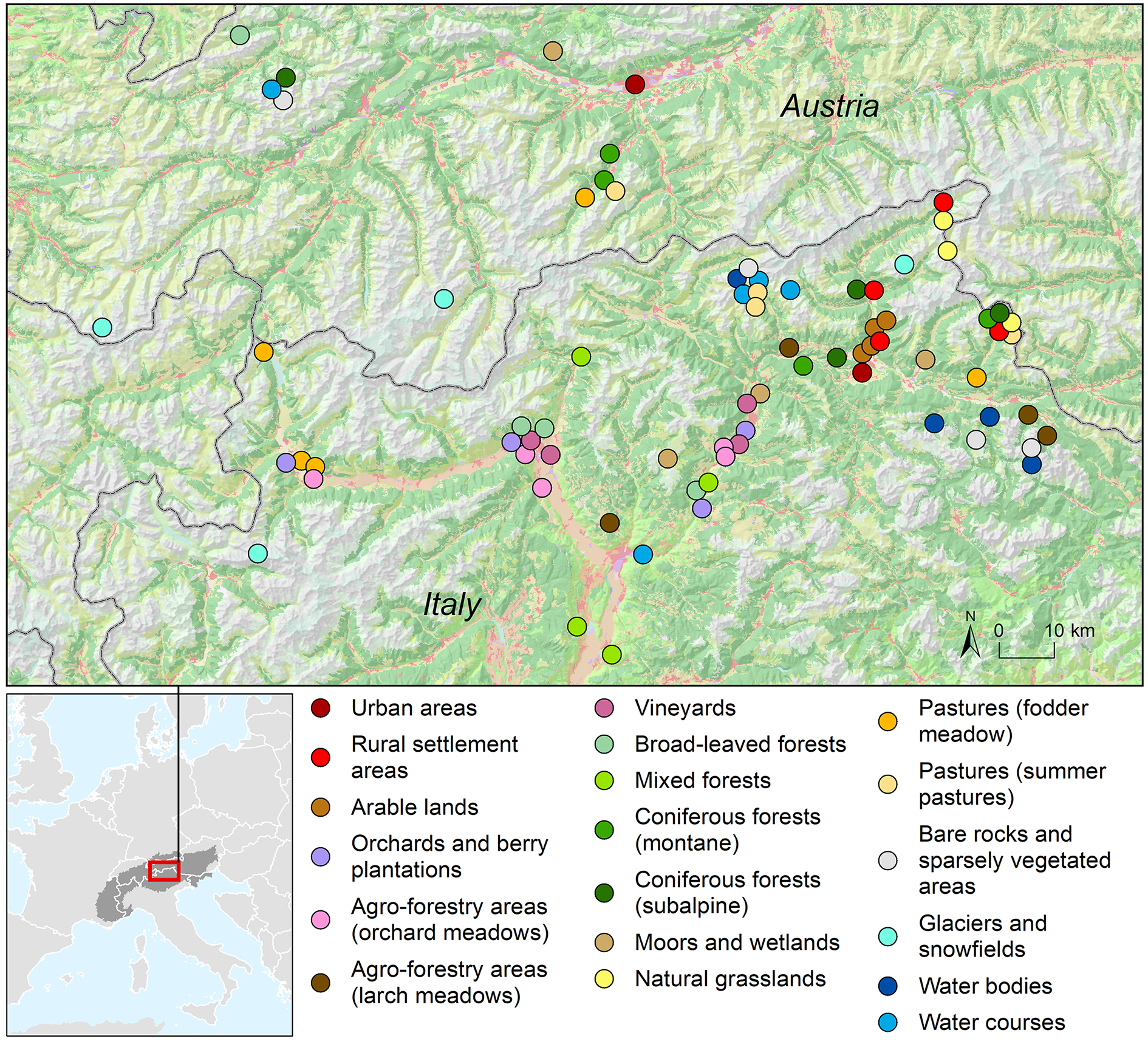

Supplement: S1 Fig — Data sources: EEA (2016; 2019) and OpenStreetMap (https://www.openstreetmap.org). (TIF) [file pone.0273519.s001.tif]

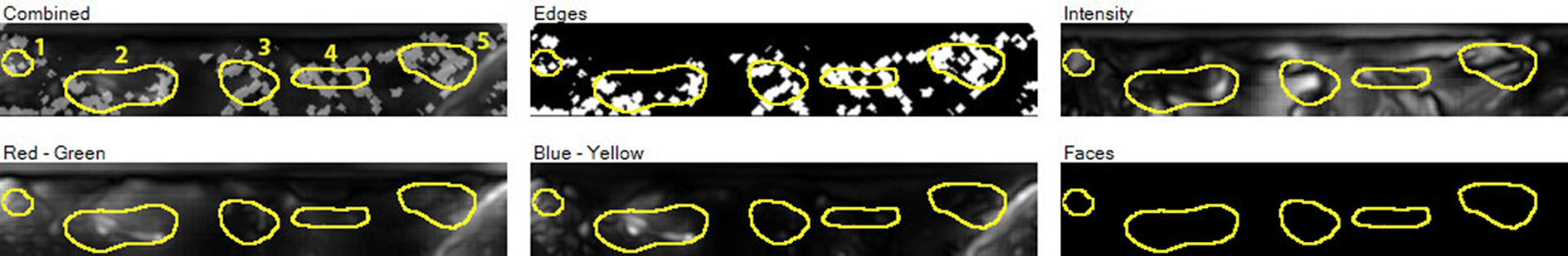

Supplement: S2 Fig — The yellow circles indicate the hotspots identified in 3M-VAS, which were used for estimating the importance of each visual element within each hotspot on a scale of from 0 to 100%. For example, for hotspot no. 3, edges take up 50% of the area, intensity has mostly medium to high values (grey to light grey areas), red-green contrast values are low (dark grey patterns) and blue-yellow contrasts as well as no values for faces (black) are missing. Estimated contributions are 50% for edges, 40% for intensity, 5% for red-green color contrast and 0% for blue-yellow color contrast and faces. (TIF) [file pone.0273519.s002.tif]
