# Supplementary material for: Potential of eye-tracking simulation software for analyzing landscape preferences
Source: PLoS One. 2022 Oct 27;17(10):e0273519. doi: 10.1371/journal.pone.0273519 (PMC9612490; doi:10.1371/journal.pone.0273519)
Supplement: S1 Table — (PDF) [file pone.0273519.s003.pdf]

S1 Table. Input data for GIS-based landscape analysis according to Schirpke et al. (2021).

Distance zone	Spatial resolution	Dataset	Data source
1: At observer point (0-60m)	1°x1° (~27.4 x 27.4 m)	Habitat map with 95 habitat types; original mapping scale 1:25,000	Own mapping according to Tasser et al. (2009)
2: Near zone: 0-1.5 km	1°x1° (~27.4 x 27.4 m)	Digital elevation model (DEM)	EEA (2016)
		Habitat map with 95 habitat types; original mapping scale 1:25,000	Own mapping according to Tasser et al. (2009)
3: Middle zone: 1.5-10 km	100x100 m	Digital elevation model (DEM)	EEA (2016)
		CORINE land cover 2012 Version 18 (09/2016) Aggregated into six classes: forest, grassland, settlement, rock, water and glacier	EEA (2019)
4: Far zone: 10-50 km	1x1km	Digital elevation model (DEM)	EEA (2016)
		CORINE land cover 2012 Version 18 (09/2016) Aggregated into five classes: forest, grassland, settlement, water and glacier	EEA (2019)

European Environment Agency (EEA), 2019. CORINE Land Cover (CLC) 2012, Version 18.5.1. http://land.copernicus.eu/pan-european/corine-land-cover/clc-2012 (accessed 28.03.22).

European Environmental Agency (EEA), 2016. European Digital Elevation Model (EU-DEM), Version 1.0. https://land.copernicus.eu/imagery-in-situ/eu-dem/eu-dem-v1-0-and-derived-products/eu-dem-v1.0 (accessed 28.03.22).

Schirpke, U., Zoderer, B.M., Tappeiner, U., Tasser, E., 2021. Effects of past landscape changes on aesthetic landscape values in the European Alps. Landscape and Urban Planning 212, 104109. https://doi.org/10.1016/j.landurbplan.2021.104109

E. Tasser, F.V. Ruffini, U. Tappeiner. 2009. An integrative approach for analysing landscape dynamics in diverse cultivated and natural mountain areas. Landscape Ecology, 24, 611-628. https://doi.org/10.1007/s10980-009-9337-9
