# Supplementary material for: Potential of eye-tracking simulation software for analyzing landscape preferences
Source: PLoS One. 2022 Oct 27;17(10):e0273519. doi: 10.1371/journal.pone.0273519 (PMC9612490; doi:10.1371/journal.pone.0273519)
Supplement: S2 Table — (PDF) [file pone.0273519.s004.pdf]

S2 Table. Mean preference values of 19 LULC types derived from three surveys as indicated by Schirpke et al. (2021).

LULC type	CLC Code	Preferences ($\bar{x}$)	Preferences (s.d.)	Preferences (s.e.)	Photos (n)	Respondents (n)
Urban areas	111, 121	3.76	1.27	0.90	2	768
Rural settlement areas	112, 122, 123, 124, 141, 142	6.18	0.87	0.39	5	3,669
Arable lands	210	6.22	0.30	0.15	4	1,536
Vineyards	221	5.33	0.97	0.37	7	82
Orchards and berry plantations	222	5.30	1.12	0.35	9	1,446
Agro-forestry area (orchard meadows)	240	6.52	0.82	0.27	9	100
Pastures (fodder meadows)	231	6.96	0.56	0.20	7	3,333
Pastures (summer pastures)	231	8.04	0.29	0.09	8	4,684
Agro-forestry area (larch meadows)	240	8.32	1.46	0.73	4	48
Broad-leaved forest	311	6.42	0.95	0.25	14	1,508
Coniferous forests (subalpine)	312	7.09	0.87	0.29	8	3,344
Coniferous forests (montane)	312	6.19	1.66	0.46	13	3,018
Mixed forests	313	6.08	0.93	0.25	14	188
Natural grasslands	321	8.03	0.64	0.29	5	2,924
Moors and wetlands	322	6.91	0.94	0.42	5	1,549
Bare rocks and sparsely vegetated areas	332, 333	7.92	0.83	0.26	9	1,443
Glaciers and perpetual snowfields	335	8.72	0.47	0.21	4	1,546
Water courses	511	7.08	1.00	0.27	12	2,599
Water bodies	512	8.80	0.82	0.26	8	2,934

Schirpke, U., Zoderer, B.M., Tappeiner, U., Tasser, E., 2021. Effects of past landscape changes on aesthetic landscape values in the European Alps. Landscape and Urban Planning 212, 104109. https://doi.org/10.1016/j.landurbplan.2021.104109
