# Supplementary material for: Potential of eye-tracking simulation software for analyzing landscape preferences
Source: PLoS One. 2022 Oct 27;17(10):e0273519. doi: 10.1371/journal.pone.0273519 (PMC9612490; doi:10.1371/journal.pone.0273519)
Supplement: S3 Table — (PDF) [file pone.0273519.s005.pdf]

S3 Table. Visual photo content indicator values (mean; min-max) for photos mainly covered by different LULC types.

Photos mainly covered by…	n	Z (n)	$E_{nat}\_P_{1..n}$ (%)	$E_{art}\_P_{1..n}$ (%)	Sky (%)	Clouds (%)	Light (%)	Open soil (%)
Water bodies	4	3.3 (3-4)	1.5 (0-4)	7.5 (0-15)	24.3 (7-30)	6.3 (0-20)	2.0 (0-8)	2.5 (0-10)
Water courses	5	3.4 (3-4)	5.6 (0-22)	4.4 (0-22)	11.6 (4-20)	28.2 (1-60)	0.0 (0-0)	4.0 (0-20)
Glaciers and perpetual snowfields	4	3.5 (3-4)	2.5 (0-10)	0.3 (0-1)	35.0 (15-50)	11.3 (0-20)	1.0 (0-4)	0.0 (0-0)
Bare rocks and sparsely vegetated areas	4	3.8 (3-4)	12.5 (0-50)	1.0 (0-3)	37.5 (30-50)	18.0 (0-70)	1.5 (0-6)	0.0 (0-0)
Moors and wetlands	4	3.0 (2-4)	3.5 (0-14)	0.5 (0-2)	16.5 (4-30)	25.0 (0-60)	0.0 (0-0)	0.0 (0-0)
Natural grasslands	4	3.8 (2-5)	0.0 (0-0)	3.8 (3-4)	31.3 (15-70)	0.3 (0-1)	0.0 (0-0)	0.0 (0-0)
Coniferous forests (subalpine)	4	3.0 (2-4)	0.0 (0-0)	0.0 (0-0)	16.5 (4-30)	18.8 (0-35)	0.0 (0-0)	1.3 (0-5)
Coniferous forests (montane)	4	2.8 (2-4)	5.8 (0-12)	4.3 (0-15)	8.5 (2-20)	13.8 (0-40)	0.0 (0-0)	23.8 (0-95)
Mixed forests	4	1.0 (1-1)	23.8 (5-70)	1.0 (0-4)	12.0 (2-25)	0.0 (0-0)	0.0 (0-0)	47.5 (10-80)
Broad-leaved forest	4	1.0 (1-1)	5.0 (0-20)	0.0 (0-0)	8.8 (3-25)	0.0 (0-0)	3.8 (0-10)	45.0 (0-90)
Agro-forestry area (larch meadows)	4	2.3 (2-3)	0.5 (0-2)	1.0 (0-2)	18.8 (15-25)	18.8 (0-70)	1.3 (0-4)	0.5 (0-2)
Pastures (summer pastures)	5	3.8 (3-4)	0.0 (0-0)	0.4 (0-1)	21.6 (15-30)	21.0 (5-45)	0.0 (0-0)	0.0 (0-0)
Pastures (fodder meadows)	5	3.6 (3-4)	0.0 (0-0)	16.0 (0-55)	13.8 (4-20)	53.0 (15-70)	0.0 (0-0)	1.2 (0-4)
Agro-forestry area (orchard meadows)	5	2.6 (1-4)	2.0 (0-10)	5.8 (1-12)	17.6 (5-35)	8.0 (0-40)	8.8 (0-40)	4.0 (0-20)
Orchards and berry plantations	4	2.0 (1-3)	25.0 (0-50)	2.8 (0-8)	16.8 (1-40)	47.5 (0-90)	3.0 (0-10)	3.3 (0-8)
Vineyards	4	1.5 (1-3)	58.3 (3-100)	2.3 (1-4)	13.8 (10-20)	40.0 (0-80)	1.3 (0-5)	1.5 (0-4)
Arable lands	4	3.8 (3-4)	10.3 (2-21)	0.0 (0-0)	28.8 (20-45)	6.3 (1-15)	0.0 (0-0)	0.0 (0-0)
Rural settlement areas	4	3.3 (3-4)	0.0 (0-0)	33.8 (15-50)	23.0 (7-40)	6.8 (2-15)	0.0 (0-0)	0.0 (0-0)
Urban areas	2	3.0 (2-4)	1.0 (0-2)	70.0 (70-70)	18.5 (17-20)	5.5 (1-10)	0.0 (0-0)	5.0 (0-10)
All	4	2.9 (1-4)	8.1 (0-100)	6.3 (0-70)	19.7 (1-50)	19.7 (0-90)	1.3 (0-40)	7.1 (0-95)
