# Supplementary material for: Potential of eye-tracking simulation software for analyzing landscape preferences
Source: PLoS One. 2022 Oct 27;17(10):e0273519. doi: 10.1371/journal.pone.0273519 (PMC9612490; doi:10.1371/journal.pone.0273519)
Supplement: S4 Table — LULC 1: water bodies & courses (%); LULC 2: glaciers and snowfields (%); LULC 3: Rocks, screen slopes; LULC 4: moors and wetlands (%); LULC 5: coniferous forests (subalpine) (%); LULC 6: coniferous forests (montane) (%); LULC 7: mixed forests (%); LULC 8: broad-leaved forests (%); LULC 9: grasslands (alpine grasslands, summer pastures) (%); LULC 10: pastures (fodder meadows) (%); LULC 11: orchards and berry plantations (%); LULC 12: vineyards (%); LULC 13: arable lands (%); LULC 14: rural settlement areas (%); LULC 15: urban areas (%). (PDF) [file pone.0273519.s006.pdf]

**S4 Table.** Estimated area of visually well distinguishable LULC types (mean; min-max) within the photos.

| Photos mainly covered by… | LULC 1 | LULC 2 | LULC 3 | LULC 4 | LULC 5 | LULC 6 | LULC 7 | LULC 8 | LULC 9 | LULC 10 | LULC 11 | LULC 12 | LULC 13 | LULC 14 | LULC 15 |
|---|---|---|---|---|---|---|---|---|---|---|---|---|---|---|---|
| Water bodies | 31.3 (25-40) | 10.0 (0-35) | 22.5 (5-40) | 0.0 (0-0) | 0.0 (0-0) | 16.3 (0-35) | 0.0 (0-0) | 0.0 (0-0) | 15.0 (0-50) | 0.0 (0-0) | 0.0 (0-0) | 0.0 (0-0) | 0.0 (0-0) | 2.5 (0-10) | 0.0 (0-0) |
| Water courses | 13.0 (5-20) | 1.2 (0-5) | 26.0 (10-45) | 0.0 (0-0) | 0.0 (0-0) | 9.0 (0-35) | 0.0 (0-0) | 6.0 (0-20) | 38.8 (0-80) | 0.0 (0-0) | 0.0 (0-0) | 0.0 (0-0) | 0.0 (0-0) | 6.0 (0-30) | 0.0 (0-0) |
| Glaciers and perpetual snowfields | 0.0 (0-0) | 27.5 (20-40) | 61.3 (50-70) | 0.0 (0-0) | 0.0 0-0) | 1.3 (0-5) | 0.0 (0-0) | 0.0 (0-0) | 10.0 (0-30) | 0.0 (0-0) | 0.0 (0-0) | 0.0 (0-0) | 0.0 (0-0) | 0.0 (0-0) | 0.0 (0-0) |
| Bare rocks and sparsely vegetated areas | 0.0 (0-0) | 8.0 (0-30) | 60.8 (55-70) | 0.0 (0-0) | 0.0 (0-0) | 10.0 (0-25) | 0.0 (0-0) | 0.0 (0-0) | 21.3 (5-40) | 0.0 (0-0) | 0.0 (0-0) | 0.0 (0-0) | 0.0 (0-0) | 0.0 (0-0) | 0.0 (0-0) |
| Natural grasslands | 1.3 (0-5) | 16.3 (5-40) | 1.3 (0-5) | 0.0 (0-0) | 5.0 (0-10) | 11.3 (0-40) | 0.0 (0-0) | 0.0 (0-0) | 76.3 (55-85) | 0.0 (0-0) | 0.0 (0-0) | 0.0 (0-0) | 0.0 (0-0) | 0.0 (0-0) | 0.0 (0-0) |
| Moors and wetlands | 5.0 (0-20) | 0.0 (0-0) | 6.3 (5-10) | 35.0 (5-60) | 0.0 (0-0) | 5.0 (0-10) | 0.0 (0-0) | 31.3 (0-50) | 22.5 (0-90) | 0.0 (0-0) | 0.0 (0-0) | 0.0 (0-0) | 0.0 (0-0) | 0.0 (0-0) | 0.0 (0-0) |
| Coniferous forests (subalpine) | 0.0 (0-0) | 0.3 (0-1) | 2.5 (0-10) | 0.0 (0-0) | 72.3 (60-80) | 0.0 0-0) | 0.0 (0-0) | 0.0 (0-0) | 25.0 (10-40) | 0.0 0-0) | 0.0 (0-0) | 0.0 (0-0) | 0.0 (0-0) | 0.0 (0-0) | 0.0 (0-0) |
| Coniferous forests (montane) | 0.0 (0-0) | 0.0 (0-0) | 2.5 (0-10) | 0.0 (0-0) | 0.0 (0-0) | 86.3 (70-100) | 0.0 (0-0) | 0.0 (0-0) | 8.8 (0-20) | 1.3 (0-5) | 0.0 (0-0) | 0.0 (0-0) | 0.0 (0-0) | 1.3 (0-5) | 0.0 (0-0) |
| Mixed forests | 0.0 (0-0) | 0.0 (0-0) | 1.3 (0-5) | 0.0 (0-0) | 0.0 0-0) | 0.0 (0-0) | 95.0 (90-100) | 0.0 (0-0) | 2.5 (0-5) | 0.0 (0-0) | 0.0 (0-0) | 0.0 (0-0) | 0.0 (0-0) | 1.3 (0-5) | 0.0 (0-0) |
| Broad-leaved forest | 0.0 (0-0) | 0.0 (0-0) | 1.3 (0-5) | 0.0 (0-0) | 0.0 0-0) | 0.0 (0-0) | 0.0 (0-0) | 91.3 (80-100) | 6.3 (0-20) | 0.0 (0-0) | 0.0 (0-0) | 0.0 (0-0) | 0.0 (0-0) | 0.0 (0-0) | 0.0 (0-0) |
| Agro-forestry area (larch meadows) | 0.0 (0-0) | 0.0 (0-0) | 0.0 (0-0) | 0.0 (0-0) | 32.5 0-70) | 15.0 (0-30) | 0.0 (0-0) | 0.0 (0-0) | 33.3 (0-65) | 17.5 (0-70) | 0.0 (0-0) | 0.0 (0-0) | 0.0 (0-0) | 1.8 (0-5) | 0.0 (0-0) |
| Pastures (summer pastures) | 0.0 (0-0) | 0.0 (0-0) | 12.4 (0-30) | 0.0 (0-0) | 20.6 0-60) | 4.0 (0-20) | 0.0 (0-0) | 0.0 (0-0) | 62.8 (35-92) | 0.0 (0-0) | 0.0 (0-0) | 0.0 (0-0) | 0.0 (0-0) | 0.2 (0-1) | 0.0 (0-0) |
| Pastures (fodder meadows) | 1.0 (0-5) | 0.0 (0-0) | 1.0 (0-5) | 0.0 (0-0) | 0.0 0-0) | 24.0 (20-35) | 1.0 (0-5) | 8.0 (0-20) | 0.0 (0-0) | 50.2 (25-75) | 0.0 (0-0) | 0.0 (0-0) | 0.0 (0-0) | 14.0 (0-45) | 0.0 (0-0) |
| Agro-forestry area (orchard meadows) | 0.0 (0-0) | 0.0 (0-0) | 0.0 (0-0) | 0.0 (0-0) | 0.0 0-0) | 4.0 (0-10) | 0.0 (0-0) | 9.0 (0-30) | 10.0 (0-50) | 46.2 (0-66) | 24.0 (5-40) | 0.0 (0-0) | 0.0 (0-0) | 6.8 (0-15) | 0.0 (0-0) |
| Orchards and berry plantations | 0.0 (0-0) | 0.0 (0-0) | 0.0 (0-0) | 0.0 (0-0) | 0.0 0-0) | 1.3 (0-5) | 0.0 (0-0) | 2.5 (0-10) | 0.0 (0-0) | 7.5 (5-10) | 88.8 (80-95) | 1.3 (0-5) | 0.0 (0-0) | 0.0 (0-0) | 0.0 (0-0) |
| Vineyards | 0.0 (0-0) | 0.0 (0-0) | 0.0 (0-0) | 0.0 (0-0) | 0.0 (0-0) | 2.5 (0-10) | 0.0 (0-0) | 1.3 (0-5) | 0.0 (0-0) | 16.3 (5-30) | 0.0 (0-0) | 80.0 (70-95) | 0.0 (0-0) | 0.0 (0-0) | 0.0 (0-0) |
| Arable lands | 0.0 (0-0) | 0.0 (0-0) | 0.0 (0-0) | 0.0 (0-0) | 0.0 (0-0) | 21.3 (10-30) | 0.0 (0-0) | 8.8 (0-15) | 0.0 (0-0) | 0.0 (0-0) | 0.0 (0-0) | 0.0 (0-0) | 70.0 (60-80) | 0.0 (0-0) | 0.0 (0-0) |
| Rural settlement areas | 0.0 (0-0) | 0.0 (0-0) | 2.5 (0-5) | 0.0 (0-0) | 6.3 (0-25) | 17.5 (0-40) | 0.0 (0-0) | 10.0 (0-30) | 5.0 (0-10) | 10.0 (0-20) | 0.0 (0-0) | 0.0 (0-0) | 0.0 (0-0) | 48.8 (40-60) | 0.0 (0-0) |
| Urban areas | 0.0 (0-0) | 0.0 (0-0) | 0.0 (0-0) | 0.0 (0-0) | 0.0 (0-0) | 5.0 (0-10) | 0.0 (0-0) | 0.0 (0-0) | 0.0 (0-0) | 0.0 (0-0) | 0.0 (0-0) | 0.0 (0-0) | 0.0 (0-0) | 0.0 (0-0) | 95.0 (90-100) |
| All | 2.8 (0-40) | 2.4 (0-40) | 10.8 (0-70) | 1.8 (0-60) | 7.3 (0-80) | 12.4 (0-100) | 4.9 (0-100) | 8.9 (0-100) | 18.7 (0-92) | 8.9 (0-75) | 6.1 (0-95) | 4.2 (0-95) | 3.6 (0-80) | 4.6 (0-60) | 2.4 (0-100) |

LULC 1: water bodies & courses (%); LULC 2: glaciers and snowfields (%); LULC 3: Rocks, screen slopes; LULC 4: moors and wetlands (%); LULC 5: coniferous forests (subalpine) (%); LULC 6: coniferous forests (montane) (%); LULC 7: mixed forests (%); LULC 8: broad-leaved forests (%); LULC 9: grasslands (alpine grasslands, summer pastures) (%); LULC 10: pastures (fodder meadows) (%); LULC 11: orchards and berry plantations (%); LULC 12: vineyards (%); LULC 13: arable lands (%); LULC 14: rural settlement areas (%); LULC 15: urban areas (%).
