# Supplementary material for: Potential of eye-tracking simulation software for analyzing landscape preferences
Source: PLoS One. 2022 Oct 27;17(10):e0273519. doi: 10.1371/journal.pone.0273519 (PMC9612490; doi:10.1371/journal.pone.0273519)
Supplement: S5 Table — (PDF) [file pone.0273519.s007.pdf]

S5 Table. Landscape metrics (mean; min-max) for photos mainly covered by different LULC types.

Photos mainly covered by… (LULC type)	AREA_MD	CIRCLE_SD	LPI	MSIDI	NP	PR	PD	CONTIG_MN	SHAPE_CV	GYRATE_MD	TA_1	TA_2
Water bodies	525.2 (158-1035)	0.2 (0.2-0.3)	22.4 (15.4-29.9)	0.6 (0.2-0.9)	61.0 (43.0-73.0)	8.0 (6.0-9.0)	1.8 (0.6-2.8)	0.6 (0.5-0.7)	41.7 (38.5-44.3)	100.6 (58-150)	143.1 (110-198)	705.0 (297-1287)
Water courses	752.8 (46-1530)	0.2 (0.2-0.2)	23.5 (9.9-44.0)	0.7 (0.4-1.0)	97.0 (77.0-113.0)	11.2 (8.0-13.0)	3.2 (0.7-9.8)	0.6 (0.5-0.7)	33.5 (29.6-38.4)	80.8 (41-126)	148.6 (112-1809)	752.4 (385-1383)
Glaciers and perpetual snowfields	2201.6 (476-3907)	0.2 (0.2-0.3)	17.2 (12.7-25.2)	0.5 (0.4-0.6)	110.8 (44-177)	7.3 (6.0-8.0)	0.6 (0.2-1.4)	0.7 (0.5-0.9)	40.9 (37.7-45.7)	281.7 (45-388)	151.8 (49-290)	2171.3 (1065-3863)
Bare rocks and sparsely vegetated areas	1389.5 (357-2333)	0.2 (0.2-0.3)	26.5 (9.8-46.8)	0.6 (0.2-0.7)	87.8 (30-140)	8.3 (5.0-11.0)	1.3 (0.2-4.1)	0.7 (0.6-0.9)	36.7 (33.1-41.3)	242.9 (97-388)	82.0 (9-152)	1418.7 (638-2690)
Natural grasslands	650.9 (218-956)	0.2 (0.2-0.2)	13.3 (9.8-16.4)	0.9 (0.8-1.0)	80.8 (42-95)	11.8 (9.0-13.0)	1.0 (0.7-1.7)	0.7 (0.7-0.8)	40.0 (37.2-42.6)	130.6 (97-204)	93.4 (57-130)	2377.8 (1479-3120)
Moors and wetlands	1328.6 (452-3240)	0.2 (0.2-0.2)	24.3 (15.2-37.6)	0.7 (0.5-0.8)	118.3 (99-135)	10.5 (8.0-14.0)	1.3 (0.5-1.9)	0.7 (0.6-0.8)	40.7 (37.2-43.8)	104.2 (80-139)	144.3 (27-218)	2347.5 (1117-4109)
Coniferous forests (subalpine)	584.2 (0-1482)	0.2 (0.0-0.3)	15.8 (0.0-27.8)	0.6 (0.0-0.9)	72.5 (0-126)	9.0 (0.0-16.0)	1.3 (0.0-2.5)	0.5 (0.0-0.8)	33.1 (0.0-49.3)	91.5 (0-232)	128.0 (0-278)	1479.5 (0-2699)
Coniferous forests (montane)	234.8 (0-939)	0.0 (0.0-0.2)	2.8 (0.0-11.2)	0.2 (0.0-0.8)	25.8 (0-103)	2.0 (0.0-8.0)	0.2 (0.0-0.6)	0.2 (0.0-0.8)	13.2 (0.0-52.6)	63.0 (0-252)	0.4 (0-2)	1073.3 (0-4293)
Mixed forests	0.0 (0-0)	0.0 (0.0-0.0)	0.0 (0.0-0.0)	0.0 (0.0-0.0)	0.0 (0-0)	0.0 (0.0-0.0)	0.0 (0.0-0.0)	0.0 (0.0-0.0)	0.0 (0.0-0.0)	0.0 (0-0)	0.0 (0-0)	0.0 (0-0)
Broad-leaved forest	0.0 (0-0)	0.0 (0.0-0.0)	0.0 (0.0-0.0)	0.0 (0.0-0.0)	0.0 (0-0)	0.0 (0.0-0.0)	0.0 (0.0-0.0)	0.0 (0.0-0.0)	0.0 (0.0-0.0)	0.0 (0-0)	0.0 (0-0)	0.0 (0-0)
Agro-forestry area (larch meadows)	0.0 (0-0)	0.0 (0.0-0.0)	0.0 (0.0-0.0)	0.0 (0.0-0.0)	0.0 (0-0)	0.0 (0.0-0.0)	0.0 (0.0-0.0)	0.0 (0.0-0.0)	0.0 (0.0-0.0)	0.0 (0-0)	0.0 (0-0)	0.0 (0-0)
Pastures (summer pastures)	559.2 (100-944)	0.2 (0.2-0.2)	20.0 (11.8-36.8)	0.9 (0.7-1.1)	88.2 (77-111)	11.6 (10.0-15.0)	3.6 (0.7-12.4)	0.6 (0.5-0.8)	36.6 (32.5-41.4)	109.7 (41-205)	87.1 (13-154)	1384.8 (440-3726)
Pastures (fodder meadows)	641.6 (84-1254)	0.2 (0.2-0.2)	16.3 (11.2-22.6)	1.0 (0.7-1.5)	119.4 (82-164)	9.8 (8.0-13.0)	2.4 (0.6-7.2)	0.7 (0.6-0.8)	42.9 (38.3-46.5)	120.1 (54-166)	116.6 (52-225)	4566.2 (1048-8466)
Agro-forestry area (orchard meadows)	0.0 (0-0)	0.0 (0.0-0.0)	0.0 (0.0-0.0)	0.0 (0.0-0.0)	0.0 (0-0)	0.0 (0.0-0.0)	0.0 (0.0-0.0)	0.0 (0.0-0.0)	0.0 (0.0-0.0)	0.0 (0-0)	0.0 (0-0)	0.0 (0-0)
Orchards and berry plantations	0.0 (0-0)	0.0 (0.0-0.0)	0.0 (0.0-0.0)	0.0 (0.0-0.0)	0.0 (0-0)	0.0 (0.0-0.0)	0.0 (0.0-0.0)	0.0 (0.0-0.0)	0.0 (0.0-0.0)	0.0 (0-0)	0.0 (0-0)	0.0 (0-0)
Vineyards	0.0 (0-0)	0.0 (0.0-0.0)	0.0 (0.0-0.0)	0.0 (0.0-0.0)	0.0 (0-0)	0.0 (0.0-0.0)	0.0 (0.0-0.0)	0.0 (0.0-0.0)	0.0 (0.0-0.0)	0.0 (0-0)	0.0 (0-0)	0.0 (0-0)
Arable lands	732.3 (601-994)	0.2 (0.2-0.2)	21.8 (18.0-24.9)	0.8 (0.6-0.9)	109.0 (98-125)	10.8 (9.0-12.0)	1.6 (1.3-1.8)	0.7 (0.6-0.7)	49.8 (44.3-57.1)	96.7 (72-139)	290.8 (267-316)	2840.8 (1803-4406)
Rural settlement areas	462.8 (63-728)	0.2 (0.2-0.3)	17.8 (11.5-30.5)	0.9 (0.7-1.4)	101.3 (70-118)	10.8 (8.0-12.0)	3.3 (0.6-8.4)	0.6 (0.5-0.8)	43.9 (41.4-46.2)	127.0 (63-282)	213.5 (16-371)	2286.3 (1170-3539)
Urban areas	0.0 (0-0)	0.0 (0.0-0.0)	0.0 (0.0-0.0)	0.0 (0.0-0.0)	0.0 (0-0)	0.0 (0.0-0.0)	0.0 (0.0-0.0)	0.0 (0.0-0.0)	0.0 (0.0-0.0)	0.0 (0-0)	0.0 (0-0)	0.0 (0-0)
All	541.1 (0-3907)	0.1 (0.0-0.3)	12.1 (0.0-46.8)	0.5 (0.0-1.5)	58.9 (0-177)	6.1 (0.0-16.0)	1.2 (0.0-12.4)	0.4 (0.0-0.9)	24.7 (0.0-57.1)	83.4 (0-388)	86.6 (0-371)	1280.3 (0-8466)
