# Supplementary material for: Potential of eye-tracking simulation software for analyzing landscape preferences
Source: PLoS One. 2022 Oct 27;17(10):e0273519. doi: 10.1371/journal.pone.0273519 (PMC9612490; doi:10.1371/journal.pone.0273519)
Supplement: Attachment — Submitted filename: Review 1.pdf [file pone.0273519.s008.pdf]

PLOS ONE

Potential of eye-tracking simulation software for analysing landscape preferences

-----Review-----

Manuscript Number	PONE-D-22-12355
Article Type	Research Article
Full Title	Potential of eye-tracking simulation software for analysing landscape preferences
Short Title	Eye-tracking simulation software in landscape analysis
Keywords	mountain landscape; landscape preferences; eye-tracking simulation

The study tries to integrate eye-tracking simulation software into research on landscape preferences. Thereby the authors focus on the type of information that can be obtained through eye-tracking simulation and how this information can contribute to explain landscape preferences. In the end of the manuscript they conclude that you cannot declare landscape preferences this way.

The study convinces by a substantial combination of different methods and data. Everything is methodically collected cleanly and well brought together. But there is also some room for improvement.
Thus, the theoretical and methodological classification of the method should already start more clearly in the theoretical framework of this manuscript, including bottom up and top down processes in visual attention and locating the method of the 3M-VAS on initial attention at an early stage. A closer approximation to eye tracking methodology should also be sought in the method. Both orientations (methodological and theoretical) will help to remedy the weaknesses of the results and their interpretations.

Overall, I advise a makeover with major revisions to the manuscript.
More minor revisions are attached below.

Detailed comments, questions and suggestions	
**page, line**	**notes**
7, 141-143	In order to get the same range of the scale the data from one preference scale was rescaled from a 5- to a 10- point scale.<br>Did you check for consistencies and did you compare the distributions of the measurements? There might be differences for the measurements that are based simply on the number of scale points. Especially reliability might be an issue regarding measurements with different numbers of scale points (see e.g. Weathers, Sharma, & Niedrich, 2005, https://doi.org/10.1016/ j.jbusres.2004.08.002 or Weijters, Cabooter, & Schillewaert, 2010, https://doi.org/10. 1016/j.ijresmar.2010.02.004). Especially since the data comes from different surveys, reliability might be one of the major problems.<br>How did you cope with this issue? What did you do? Did you perform any additional analysis? If so, please report on it.
7, 143-145	The surveys were carried out between 2011 and 2019, including residents and visitors, accounting for demographics and origin.<br>Did you control for the influence of origin and sociodemographics?
7, 149-150	Since the preferences of the landscapes serve as dependent variables the selection of the stimuli is central for the results of your study. Besides the selection criteria described in the text, how did you cope with a higher number of stimuli for one LULC type if all other criteria were fulfilled? Which pictures did you choose? Were the stimuli random or systematically chosen? Please add some more detail in this paragraph.
7, 155	Please change 'Eye tracking analysis' into 'eye tracking simulation' analysis.
7, 156-157	Are there any known quality standards for 3M-VAS or measures of instrument reliability or validity? Please add some more information.
7-8,157-158	Was there a standardized upload of the images? Were the images comparable in size and resolution? Please provide further information.
8, 164-165	What do the percentages in the image describe? Is this the probability of the observation? Please add more details.<br>What kind of data are these heatmaps based on? Is it comparable to time to first fixation, number of fixations, fixation duration? Please add more information in the caption to understand the figure.
9, 175-176	Eye-tracking data are often considered dependent. This means, for example, that the emergence of a 'hotspot' through prolonged observation also minimizes the emergence of other hotspots. What about simulated data? Shouldn't they also be considered dependent on each other? And does this raise a problem for the analysis with methods for independent data?
10, 182-188	The proportion of clearly recognizable human structures, proportion of the sky, cloud cover and areas with special lighting conditions were estimated. Who estimated it? Were there coders? Were they trained, coached? According to which rules was the estimate made? Is there evidence of sufficiently high reliability and time stability of this measurements/estimations? Please add more information about this estimation process.

10, 192<br>11, 210	Do you mean landscape metrics were obtained from study with reference number 10? would you be so kind and could rephrase the sentence into a more readable version.
11, 199-203	The idea behind the use of geodata is that you have the most accurate data possible about what you see in the images.<br>But doesn't that assume a little too much knowledge about what you're seeing? The authors write that only visible areas are used. But what does visible mean? Does it mean that it is recognizable by color highlighting? Or does it have to be (a) clearly visually separable/distinguishable from the surrounding area as an independent area and (b) clearly and uniquely recognizable as a specific type of area (see also table 2)? Is a berry plantation also recognizable as such in the picture? Or is it simply a separately recognizable area without knowing what exactly it is? Do the authors add information based on the method that is not available to anyone who views the panoramic images?<br>Please provide further information to justify the use of geodata and support the benefit of using it.
12 228-229	Thank you for creating the summary for all variables added to the regression. That helps to understand your model approach.<br>Combining this information with the information about the number of cases (p7, lines 149-150), you are using 78 cases/observations (pictures) in a regression with (1) 8, (2) 19, (3) 30 and in the fourth regression (4) 49 regressors.<br>Unfortunately, regression models with a high number of independent variables and a limited number of observations are prone to overfitting. Since all models have a rather unfavorable ratio of observations and explanatory variables, the problem seems to be relevant for your regression models. How did you cope with this issue? Since the sample and the complexity of the models can hardly be changed now, a clear limitation of the findings must be made in the discussion section. Maybe you are also able to reduce the complexity of the regression approach by including similar or opposite indicators (e.g., the HP_{min} HP_{max} values) in the model exclusively rather than together. This decision could be justified theoretically and statistically.<br>To proof your model, you could also add more pictures or change the pictures and corresponding data in your regression. Approximately equal estimates show a reliable model approach. Did you change the pictures/their data and tested the regression models with more or other pictures/observations? What results did you achieve? This manuscript needs more evidence of your approach at this critical point. Add more information, also for cross validation of your models!
18, 323-327	But there was the opportunity to gather some of these data about single elements using the unused reports from 3M-VAS (see p8, lines 161-162). Do you think there is more potential for using the additional outputs of the software? Is the analysis of this data too complicated to use? What was your experience?<br>Couldn't this information have been collected via content analysis?
18, 331-332	One ‘)’ is missing somewhere.

18, 327-336	Thank you for this clear and quite concise summary of the weaknesses of the 3M-VAS method used. Unfortunately, the combination of (a) the limitation to "first glance" analysis combined with (b) the intentional exclusion of particularly salient visual stimuli such as lighting, shadows, etc. is a factor that severely minimizes the range of your results and maximizes the limitations of your study.<br>In eye-tracking experiments on initial visual attention, the effects of salient stimuli are often tested. By minimizing salient stimuli, a break has arisen not only methodologically, but especially theoretically. This is expressed by the fact that it is not possible to say exactly which situation is now 'imitated' using the 3M-VAS data. It is not clear which (real) situation can be represented and analyzed with the simulated data.<br>In order to draw a clearer picture of the scope of the findings, the discussion should be intensified.<br>Also in context -lines 337-344 you should consider expanding the theory part of the paper a bit especially with regard to top down and bottom up processes of visual attention. This could also provide a clearer picture in the results and discussion sections. This would also contribute to a clearer classification of the findings
20, 388-389	Regarding the mentioned points above it remains unclear if the evaluation of different scenarios by eye-tracking simulation really could provide important information for decision-making and landscape planning. In which planning or decision-making situation can the simulation of initial attention really alone or in combination provide sufficient evidence for highly complex decisions that are based not only on visual markers but also on economic, ecological or even other societal concerns on a meso or macro level?<br>Please provide less abstract and more concrete ideas for using the findings of this study in real-world decision-making situations.
20, 390-397	Just like hearing, the combination of smelling and seeing or even feeling and seeing leads to a different perception. This perspective is of a more general nature and does not contribute anything to this specific study. Therefore, I suggest shortening or omit this discussion thread.
